# Expression of HA1 antigen of H5N1 influenza virus as a potent candidate for vaccine in bacterial system

**Published:** 2016

**Authors:** A. S. Farsad, S. Malekzadeh-Shafaroudi, N. Moshtaghi, F. Fotouhi, S. Zibaee

**Affiliations:** 1 Ph.D. Student in Plant Biotechnology, Department of Biotechnology and Plant Breeding, Faculty of Agriculture, Ferdowsi University of Mashhad, Mashhad, Iran;; 2 Department of Biotechnology and Plant Breeding, Faculty of Agriculture, Ferdowsi University of Mashhad, Mashhad, Iran;; 3 Influenza Research Lab, Pasteur Institute of Iran, Tehran, Iran;; 4 Razi Vaccine and Serum Research Institute, Mashhad, Iran

**Keywords:** Avian influenza, *Escherichia coli*, HA1, Recombinant DNA, Subunit vaccine

## Abstract

The impending influenza virus pandemic requires global vaccination to prevent large-scale mortality and morbidity, but traditional influenza virus vaccine production is too slow for rapid responses. In this study, bacterial system has been developed for expression and purification of properly folded HA1 antigen as a rapid response to emerging pandemic strains. Here, a recombinant H5N1 (A/Indonesia/05/05) hemagglutinin globular domain, the synthesized HA1 (1-320 amino acids), was amplified and cloned into pET-28a bacterial expression vector. Then, his-tagged HA1 protein was expressed in *Escherichia coli* BL21 under 1 mM IPTG induction. The protein expression was optimized under a time-course induction study and further purified using Ni-NTA chromatography. Migration size of protein was detected at 40 KDa by Western blot using anti-His tag monoclonal antibody and demonstrated no discrepancy compared to its calculated molecular weight. Since most antigenic sites are in the HA1 domain of HA, using this domain of influenza virus as antigen is of great importance in vaccine development. The ability of the antibody stimulation against HA1 expressed in bacterial cells is also examined using enzyme-linked immunosorbent assay (ELISA) analysis. Upon immunization of rabbits, oligomeric HA1 elicited potent neutralizing antibodies and high levels of serum antibody binding to HA1. Our findings suggest that HA1-based vaccines can be produced efficiently in bacterial systems and can be easily upscaled in response to a pandemic influenza virus threat.

## Introduction

The recent global spread of H5N1 highlighted the need for rapid development of effective vaccines against pandemic influenza viruses (Swayne et al., 2000 ▶). H5N1 was the main virus that caused outbreak in poultry and reported human fatality in Hong Kong in 1997 (Olsen et al., 2006 ▶; Treanor et al., 2006 ▶).

 Some of the strategies being explored for develop-ment of H5N1 vaccines include inactivated whole virus, live attenuated virus, DNA vaccine, adenovirus vectored HA, recombinant antigens purified from baculovirus insect cell system and plants (Johansson and Brett, 2007 ▶; Lin et al., 2008 ▶; Shoji et al., 2009 ▶, 2011) ▶. One of the strategies is the recombinant protein expression in *E. coli*, which is simple, fast, cost effective, robust with the maximal amount yielded, and has been used in several H5N1 studies (Shen et al., 2008 ▶; Biesova et al., 2009 ▶; Chiu et al., 2009 ▶; Khurana et al., 2011a ▶). HA1, a major antigenic envelope protein, is the main focus of influenza vaccines development (Kanekiyo et al., 2013 ▶; Dilillo et al., 2014 ▶). The HA1 antigenic domain of HA has been shown to induce an immune response equal to that of the full-size protein (Tonegawa et al., 2003 ▶).

 In this study, we present the methods of construction, expression and purification of the HA1 antigen from H5N1 in bacterial expression system, *E. coli*. As protein expression using bacteria will be cheaper, the aim of this study was to determine if bacterially expressed HA1 antigens can stimulate neutralizing antibodies or not.

## Materials and Methods

**Bioinformatic analysis of HA1 antigen**

 Physio-biochemical characteristics of HA1 antigen including isoelectric point, theoretical molecular weight, aliphatic index and GRAVY were analyzed through EXPASY (ProtParam tool). The tertiary structure of HA1 protein was also determined through phyre2 protein prediction site.

**Cloning of the recombinant HA1 protein**

 The HA1 sequence, encompassing amino acids 1-320 of the A/Indonesia/05/05 strain of H5N1 influenza virus (AFM78567.1), was optimized for expression in *E. coli* by GENSCRIPT (www.Genscript.com) and synthesized by GENERAY. Start codon (AUG) and stop codon (UAA) were added into the 5´ and 3´ ends of the construct, respectively and recognition sites of BamHI and XhoI restriction enzymes were introduced into the 5´ and 3´ ends of the synthetic gene, respectively. A poly-histidine affinity purification tag (6His) was also added to the C-terminus. The synthesized sequence was removed from the pUC57 vector by digestion with BamHI and XhoI and then inserted into the linearized pET28a (+) expression vector (Novagen, USA) using T4 DNA ligase (Fermentas, USA), yielding pET28a-HA1 vector. The resulting vector transformed into *E. coli* strain DH5α cells through heat shock transformation method and they were selected on LB agar plate containing 50 µg.m^-1^ ampicillin. The selected clones were screened by restriction analysis, PCR with gene specific primers (Table 1) and verified by DNA sequencing. They subsequently transformed into *E. coli* strain BL21 (DE3) for expression analysis.

**Table 1:** Oligonucleotide primers used to PCR

Forward primer	5´ TAATGGACAATCTGGAAGAATGGAA 3´
Riverse primer	5´ GAAGTTCATCTTTTTCTCTTTGTGG 3´

**Protein expression and optimization**

*Escherichia coli* strain BL21 (DE3) cells harboring plasmids were streaked on LB agar plate supplemented with 100 µg ml^-1^ kanamycin and cultured overnight at 37°C. A single colony of bacteria was inoculated into 10 ml LB broth and incubated agitating overnight at 37°C. The LB medium (10 ml) was added with 100 µL overnight bacterial culture and incubated agitating at 37°C. At an OD600 of = 0.5, 1 mM of Isopropyl β-D-1-thiogalactopyranoside (IPTG) at final concentration was added to induce the protein expression, and incubated agitating was continued and collected at 0-6 h. The different concentrations of IPTG (0, 0.2, 0.4, 0.8 and 1 mM) were also tested at the best analysed time. After expression, the harvested cells were washed three times with ice-cold PBS (2.7 mM KCl, 137 mM NaCl, pH = 7.4) and 1% Triton X-100. Inclusion bodies were isolated by cell lysis in urea lysis buffer (7 M urea, 20 mM HEPES [4–(2–hydroxyethyl)–1–piperazineethane-sulfonic acid], pH = 7). The cells were frozen at -80°C overnight and vortexed vigorously. Supernatant was collected after centrifugation at 9000 g for 30 min at 4°C (Wei et al., 2014 ▶).

**Stepwise protein elution and purification**

 Prior to large-scale protein purification, a small-scale stepwise protein elution study was used to determine the stringency of wash condition using a gradient concen-tration of imidazole buffers. An amount of 600 μL of lysis buffer (7 M urea, 100 mM NaH_2_PO_4_, 100 mM Tris-Cl, 300 mM NaCl, pH = 8.0) was used to equilibrate Ni-NTA Spin Column (QIAGEN, Germany) and cen-trifuged for 2 min at 890 g. Then, 600 μL of the cleared lysate supernatant was loaded onto a pre-equilibrated spin column and centrifuged for 5 min at 270 × g. Flowthrough was collected. The spin column was washed with 200 μL of wash buffer (8 M urea, 100 mM NaH_2_PO_4_, 100 mM Tris-Cl, 300 mM NaCl, pH = 8.0) containing 100 mM imidazole and centrifuged for 2 min at 890 × g. The eluted fraction was collected. The wash and centrifugation steps were repeated with wash buffers containing 200, 300, 400 and 500 mM imidazole, subsequently (Khurana et al., 2011a ▶; Wei et al., 2014 ▶). All collected samples were analyzed by 12% SDS-PAGE.

 In order to obtain the purified protein, we employed nickel chromatography to isolate polyhistidine-tagged protein from bacterial contaminant proteins due to the high specific affinity of the Ni-NTA resins for histidine residues (Janknecht et al., 1991 ▶). For protein purifica-tion, a total amount of 5 ml bacterial cleared cell lysate was loaded on 1 ml bed volume of pre equilibrated nickel-nitrilotriacetic acid agarose, Ni-NTA (QIAGEN, Germany) and flowthrough was collected. The column was then washed with 10 ml wash buffer (30 mM imidazole, 8 M urea, 100 mM NaH_2_PO_4_, 100 mM Tris-Cl, 300 mM NaCl, pH = 8.0) and wash fraction was collected. Purified protein was eluted 4 times with 500 μL of elution buffer (500 mM imidazole, 8 M urea, 100 mM NaH_2_PO_4_, 100 mM Tris-Cl, 300 mM NaCl, pH = 8.0) and all fractions were collected (Shen et al., 2008 ▶; Wei et al., 2014 ▶). For refolding, the solubilized protein was slowly diluted 100-fold in redox folding buffer. The renaturation protein solution was dialyzed against 8 M urea and salts to remove the denaturing agents. The dialysate was filtered through 12000 cut-off Sigma dialysis filter. The relative protein concentration was determined by the Bradford method (Bradford, 1976 ▶).

**SDS-PAGE and Western blotting**

 Sample proteins were resolved on reducing 12% SDS-PAGE and then visualized after Coomassie brilliant blue staining. For further characterization, the separated proteins on SDS-PAGE were transferred to nitrocellulose membrane by electroblotting (Biorad, USA) for 1 h room temperature at 100 V. The membrane was then blocked with blocking buffer [BSA 1%] overnight at 4°C. The membrane was washed three times with washing buffer [0.5% (v/v) Tween 20, 1 × PBS], each for 10 min at room temperature. The membrane was probed with the conjugated anti-6x His tag^®^ mouse monoclonal antibody (Sigma-Aldrich) for 1 h and washed again three times with washing buffer. The protein bounds were visualized by staining the membrane with DAB (Diaminobenzi-dine) (Sigma–Aldrich).

**Immunization of rabbits**

 Female New Zealand White rabbits (five per group) received intramuscular (IM) injections of HA1 (90 μg), on study days 0 and 21, with chitosan adjuvant (Sigma) containing 1:10 (w:w) of chitosan. Animals in the control group received PBS with chitosan alone. Serum HA1 antibody responses were evaluated in samples collected on days 0 (prior to vaccination), 21 (prior to 2nd vaccination) and 42 (post-2nd vaccination) by enzyme-linked immunosorbent assay (ELISA) test (data not shown). Plasma was collected from immunized rabbits at day 42, defibrinated and serum separated. Rabbit sera (diluted 1:10) obtained the following two immunizations either with subunit H5N1 vaccine as positive control or with oligomeric HA1 evaluated by ELISA test (Khurana et al., 2011a ▶; Verma et al., 2012 ▶).

**Enzyme-linked immunosorbent assay**

 The resultant protein obtained in this research was used for further study to determine its capability to elicit the neutralizing antibodies raised in rabbit by using ELISA assay (Shen et al., 2008 ▶). Therefore, HA1 anti-body responses were evaluated by ELISA. ELISA plate was coated with bacterial HA1 antigen at 37°C for 1 h; followed by incubation with 1% bovine serum albumin (BSA) in PBS for 2 h at 37°C to prevent non-specific binding. The wells were washed by PBST/PBS and incubated with rabbit sera. The wells were washed again by PBST/PBS and incubated with conjugated anti-anti rabbit antibody at 37°C for 1 h. Wells were developed with OPD (Ortho-Phenylenediamine) substrate; the color reaction was stopped by 2 N H_2_SO_4_ and read at 490 nm of wavelength.

## Results

**Bioinformatic analysis results**

**Fig. 1:**
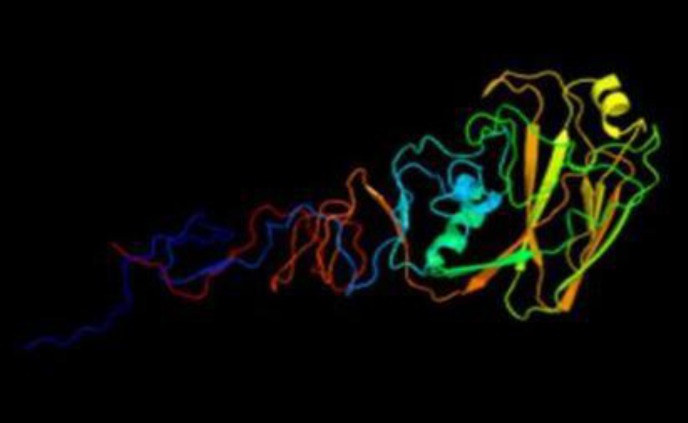
Predicted tertiary structure of HA1 protein

 Physio-biochemical studies on HA1 antigen showed that HA1 protein is stable, with isoelectric point of 6.79, theoretical molecular weight of 37 KDa, aliphatic index of 78.89 and GRAVY of -0.456. The tertiary structure of HA1 protein was also determined (Fig. 1).

**Construction and cloning of HA1 protein**

**Fig. 2:**
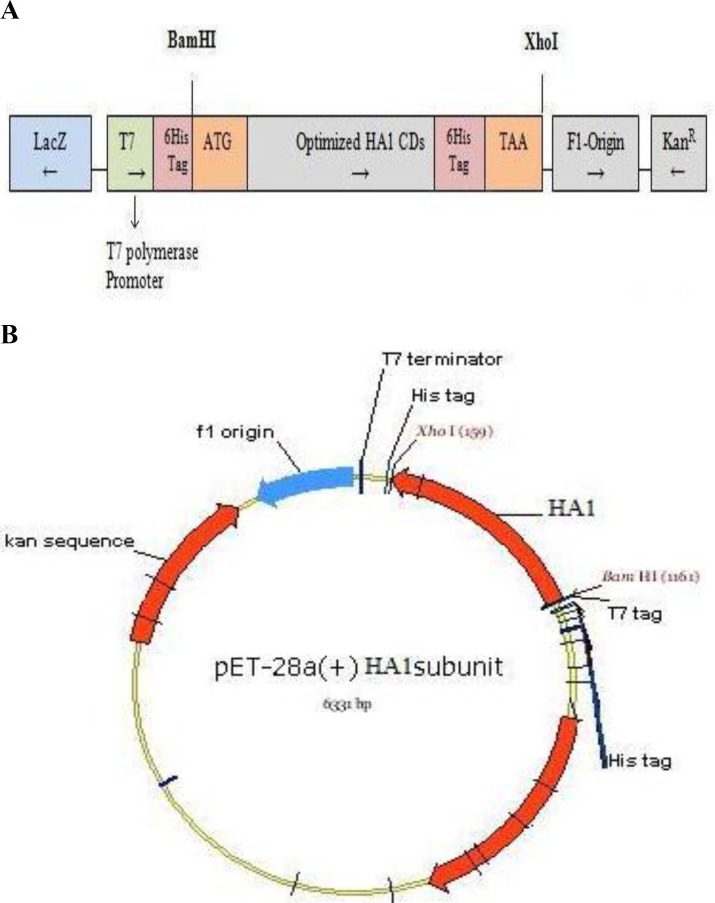
Cloning of HA1 cDNA into pET28a. (A) Schematic illustration of pET28a-HA1 construct contained optimized HA1 antigen. (B) Construct map of recombinant pET28a-HA1 vector

**Fig. 3:**
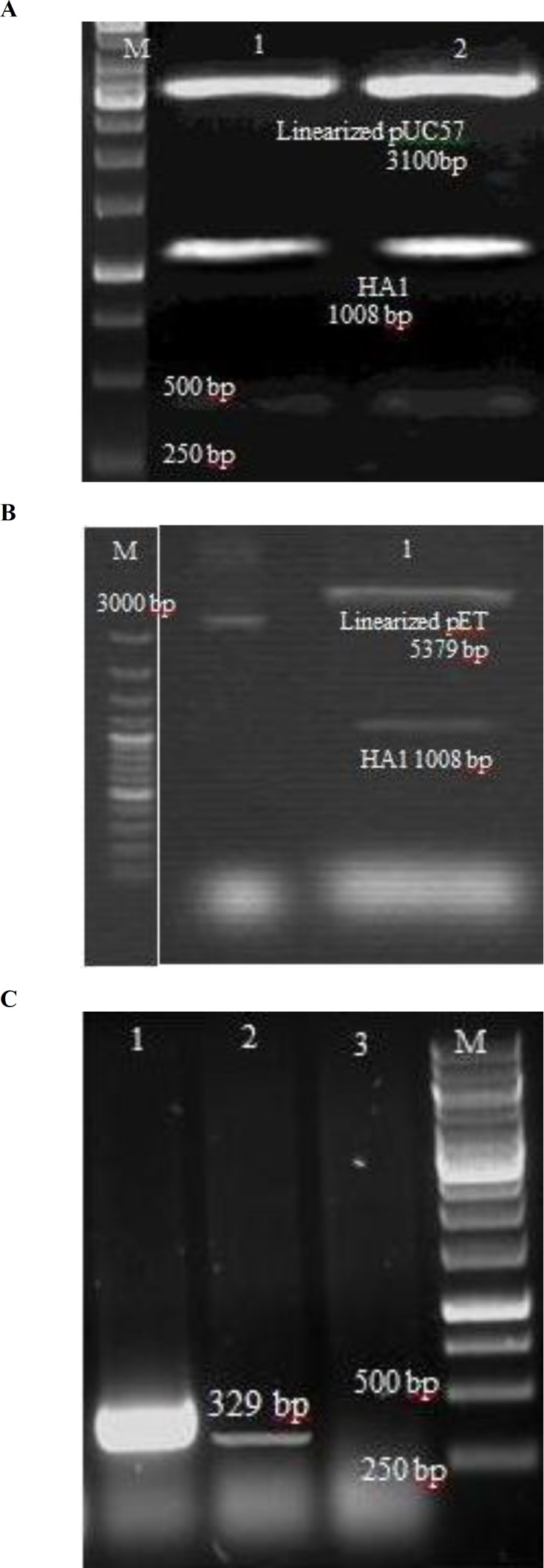
Construction and cloning of HA1 protein. (A) Digestion of pUC57 vector containing HA1 gene by enzymes BamHI and XhoI. M: Molecular marker. Lanes 1 and 2: Digested plasmid and HA1 gene. (B) Digestion of pET28a-HA1 vector by enzymes BamHI and XhoI. M: Molecular marker. Lane 1: Digested pET and HA1 gene. (C) PCR analysis for detection of HA1 gene in transformed *E. coli* clonies contained pET28a-HA1 construct by HA1 specific primers. M: Molecular marker. Lane 1: Positive control, Lane 2: Transformed bacterial clony (The 329 bp band was clear), and Lane 3: Negative control

 To evaluate the efficacy of recombinant HA1 proteins as potential vaccine candidates, cDNA encoding the HA1 was cloned into pET28a (Figs. 2A and B). Restriction analysis of cloned HA1 gene in pUC57 vector was shown in Fig. 3A. Moreover, the results of restriction analysis of infected *E. coli* with pET28a harboring HA1 and PCR assay were shown, respectively, in Figs. 3B and C. DNA sequencing analysis confirmed that the cloned gene contained no alteration and in-frame to the expression vector (data not shown).

**Optimizing expression of HA1 protein**

**Fig. 4:**
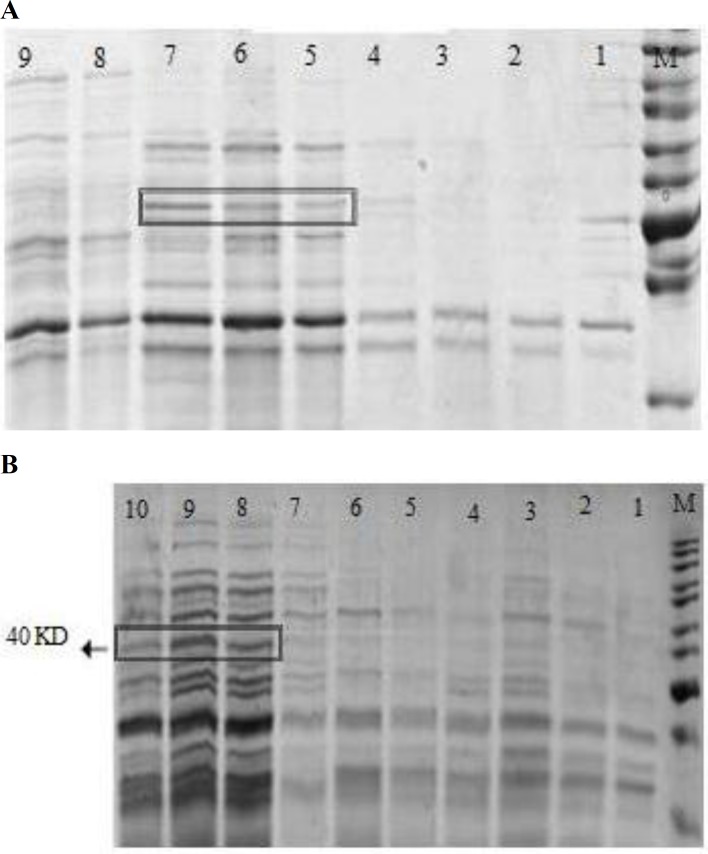
Optimizing the expression of HA1 protein. (A) Time-course induction study in *Escherichia coli* strain BL21 (DE3). Expression of HA1 protein was induced by 1 mM IPTG. Lane M: Protein ladder. Lanes 1-7: Harvested cell aggregate (*E. coli* BL21 pet28a-HA1) at 0 h, 1 h, 2 h, 3 h, 4 h, 5 h and 6 h, respectively; Lanes 8 and 9: Negative control samples (*E. coli* BL21 pet28a+) at 0 h and 6 h, respectively. (B) The best IPTG concentration study after 6 h induction. Lane M: Protein ladder. Lanes 1-5: Control samples (*E. coli* BL21 pet28a+) with 0, 0.2, 0.4, 0.8 and 1 Mm IPTG concentration, respectively. Lane 6-10: HA1 expressed samples (*E. coli* BL21 pet28a-HA1) with 0, 0.2, 0.8, 1 and 0.4 mM IPTG concentration, respectively

 Blotting results of HA1 protein in *E. coli* BL21 were shown in Fig. 4A, verifying that a highest level expression of HA1 protein was obtained at 6 h of IPTG-induction at 37°C and the best IPTG concentration after 6 h induction was obtained at 1 mM (Fig. 4B). Therefore, the small-scale time-course induction study confirmed that the maximal yield of protein was obtained at 6 h of induction.

**Protein purification and optimization**

**Fig. 5:**
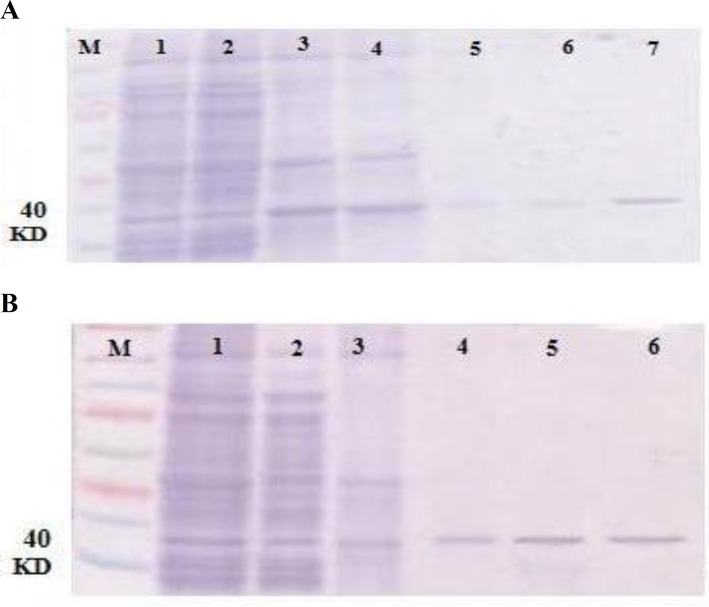
SDS-PAGE gel analysis of the purified HA1 protein. (A) 12% SDS-PAGE gel analysis of stepwise elution of HA1 protein. Protein was eluted with a stepwise gradient of 100-500 mM imidazole. Lane M: Pre-stained protein ladder. Lane 1: Cleared lysate; Lane 2: Flow through fraction; Lanes 3-7: Eluted fractions (100, 200, 300, 400, 500 mM imidazole, respectively). (B) 12% SDS-PAGE gel analysis of the large-scale HA1 protein purification. Lane M: Pre-stained protein ladder. Lane 1: Cleared lysate; Lane 2: Flow-through fraction; Lane 3: Wash fraction; Lanes 4-6: Eluted fractions (500 mM imidazole). 40 KDa band shows HA1 molecular weight

 Recombinant HA1 was expressed as inclusion bodies and extracted with 8 M urea. Our data (Fig. 5A) demonstrated 6X histidine tagged recombinant protein was eluted at 500 mM of imidazole. Thus, the large-scale purification of HA1 protein was washed with 30 mM imidazole and all purified proteins were eluted at 500 mM imidazole buffer. The purification of HA1 protein was resolved on SDS-PAGE gel (Fig. 5B). Gel analysis of protein purity confirmed that this method was feasible to obtain the high purified protein (Fig. 5B).

**Fig. 6:**
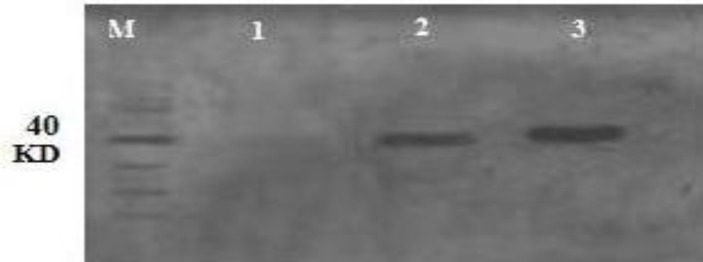
Western blotting of the HA1 protein using conjugated anti-His tag antibody in 1:1000 dilution. Lane M: Fermentase protein ladder. Lane 1: Cleared lysate of non-harbouring plasmid *E. coli* strain BL21 as negative control; Lanes 2 and 3: Eluted fractions (500 mM imidazole). 40 KDa band shows HA1 molecular weight

 Purified bacterial inclusion body proteins were subjected to SDS-PAGE for detection by Western blot analysis (Fig. 6). Using a conjugated anti-His mono-clonal antibody, recombinant proteins of expected molecular weights (HA1 (1-320 amino acid), 40 KDa) are depicted in Fig. 5. Electrophoretic migration size of denatured HA1 protein revealed no aberrant migration and they were almost identical to their theoretical molecular weight. Therefore, the bacterial expression system could produce the efficient recombinant HA1 protein for immunogenicity study.

**Immunogenicity study in rabbits**

**Fig. 7:**
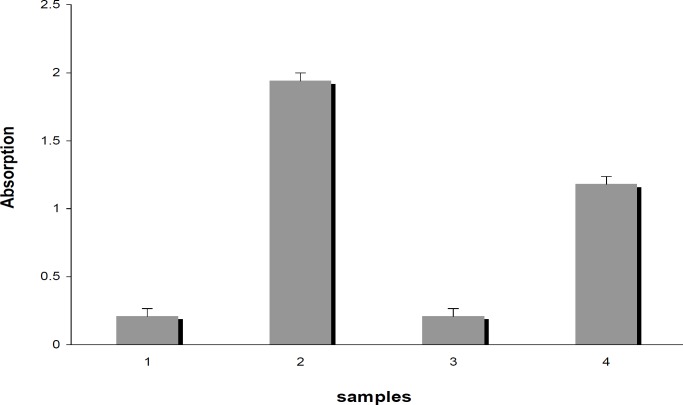
Immunogenicity study in rabbit sera using ELISA. 1 and 3: Injected rabbit sera with PBS with chitosan as negative control, 2: Injected rabbit sera with subunit H5N1 vaccine as positive control, and 4: Injected rabbit sera with bacterial HA1 protein

 Following the immunogenicity study, post-vaccination serum antibody affinity to the HA1 antigen was evaluated using ELISA test. The ELISA results demonstrated that dose of 90 μg of HA1 plus chitosan adjuvant elicited serum antibody in vaccinated rabbits in comparison to the positive and negative controls (Fig. 7). Because of showing the positive results in ELISA test, the HA1 protein expressed from bacterial system can be a potential candidate for the development of a safe and efficient vaccine for HPAI (H5N1) influenza virus.

## Discussion

 The HA1 subunit (1-320 amino acids) forms a globular head which contains the receptor binding domain (RBD), a main target for neutralizing antibodies (Stevens et al., 2006 ▶). HA1 binds to sialic acid-containing receptors on the cell surface, bringing about the attachment of the virus particle to the cell and this attachment induces virus internalization (Chiu *et al*., 2009). In order to design an effective and safe HA1-based vaccine, it is essential to cover the RBD of H5N1 HA protein in HA1 subunit (Shoji et al., 2009 ▶; Tsai et al., 2012 ▶), because RBD induces the production of neutralizing antibodies.

In recent decades, a large number of studies have been initiated to investigate the possibility to express recombinant vaccine antigens in bacterial system as they are considered as safe, low cost, easy to produce and rapid to upscale compared with traditional inactivated or live attenuated egg-based vaccines. In this study, we focused on HA1 expression as an interesting candidate for a universal vaccine against H5N1 influenza virus in *E. coli* using a simple, robust and scalable process. The recombinant protein was refolded and purified from the insoluble fraction of the cellular lysate. Recombinant HA1 appears to be properly folded, as shown by immunological assays. It could induce the specific serum antibodies and was found to be immunogenic, to be capable of triggering the production of neutralizing antibodies, and to have protective activity in the rabbit model.

 Khurana et al. (2011b) ▶ reported that the properly folded HA1 (1-320 amino acids), i.e., HA1 lacking amino acids 321 to 330, contained >75% functional oligomers without addition of foreign oligomerization signal to the first five amino acids in the N terminus of HA1. So, upon immunization of rabbits, the oligomeric HA1 elicited potent neutralizing antibodies against homologous and heterologous H5N1 viruses more rapidly than HA1 (28-320 amino acids) containing only monomers. In fact, they described the presence of an N-terminal oligomerization sequence in the globular domain of influenza virus hemagglutinin for the first time. Verma et al. (2012 ▶) also showed that oligomeric recombinant H5-HA1 vaccine produced in bacteria protects ferrets from homologous and heterologous wild-type H5N1 influenza challenge and controls viral loads better than subunit H5N1 vaccine by eliciting high-affinity antibodies. According to their findings, oligomeric HA1 (1-320 amino acids) generated more cross-neutralizing antibodies and higher levels of serum antibody binding to HA1, with stronger avidity and a better IgG/IgM ratio, than monomeric HA1. In this study, the expression and immunization of a synthesized HA1 domain of influenza H5N1 virus was investigated in bacterial system and our results confirm the findings of Khurana et al. (2011b) ▶ and Verma et al. (2012) ▶. This paper suggests that functional oligomeric HA1 proteins can be produced efficiently in bacterial systems and could provide a mechanism for rapid large-scale production of influenza vaccines in the face of influenza pandemic threat. The immunogenicity study in rabbits indicates that bacterial HA1 antigen can induce serum HA1 antibody responses in tested animals and be used for further analysis in influenza vaccine production. Expression of recombinant HA1 in bacterial system can provide a rapid and economical approach for early response to impending influenza pandemic. However, it was not known if non-glycosylated proteins in bacterial systems would present antigenically relevant epitopes. Recently, studies demonstrated that bacterially produced influenza HA1 domains (1-320 amino acids) from several pandemic strains are properly folded, form functional oligomers that can agglutinate red blood cells (RBC), and elicit broadly neutralizing antibodies upon immunization (Khurana et al. 2010 ▶, 2011b ▶).

 In summary, bacterially expressed recombinant HA1 immunogens may provide an alternative vaccine plat-form. When combined with the appropriate adjuvant, they are likely to generate antibodies with the capacity to neutralize subtype-specific influenza strains. In addition, recombinant HA1 antigen of H5N1 which was bacterially expressed and refolded *in vitro* can also be used in direct ELISA for detection and differentiation of subtype-specific antibodies.

 Taken together, the here described recombinant influenza technology allows the fast and easy production of large amounts of vaccines based on the influenza HA globular domain. This technology may be used to produce seasonal influenza vaccines as well as vaccines against newly emerging pandemic influenza outbreaks.

## References

[B1] Biesova Z, Miller MA, Schneerson R, Shiloach J, Green KY, Robbins JB, Keith JM (2009). Preparation, characterization, and immunogenicity in mice of a re-combinant influenza H5 hemagglutinin vaccine against the avian H5N1 A/Vietnam/1203/2004 influenza virus. Vaccine.

[B2] Bradford MM (1976). A rapid and sensitive method for the quantification of microgram quantities of protein utilizing the principle of protein-dye binding. Anal. Biochem.

[B3] Chiu FF, Venkatesan N, Wu CR, Chou AH, Chen HW, Lian SP, Liu SJ, Huang CC, Lian WC, Chong P, Leng CH (2009). Immunological study of HA1 domain of hemagglutinin of influenza H5N1 virus. Biochem. Biophys. Res. Commun.

[B4] Dilillo DJ, Tan GS, Palese P, Ravetch JV (2014). Broadly neutralizing hemagglutinin stalk-specific anti-bodies require Fcgamma R interactions for protection against influenza virus in vivo. Nature Med.

[B5] Janknecht R, de Martynoff G, Lou J, Hipskind RA, Nordheim A, Stunnenberg HG (1991). Rapid and efficient purification of native histidine-tagged protein expressed by recombinant vaccinia virus. Proc. Natl. Acad. Sci. U. S. A.

[B6] Johansson BE, Brett IC (2007). Changing perspective on immunization against influenza. Vaccine.

[B7] Kanekiyo M, Wei CJ, Yassine HM, McTamney PM, Boyington JC, Whittle JR, Rao SS, Kong WP, Wang L, Nabel GJ (2013). Self-assembling influenza nanoparticle vaccines elicit broadly neutralizing H1N1 antibodies.

[B8] Khurana S, Larkinb C, Vermaa S, Joshib MB, Fontanac J, Stevenc AC, Kinga LR, Manischewitza J, McCormickb W, Guptab RK, Goldinga H (2011a). Recombinant HA1 produced in E coli forms functional oligomers and generates strain-specific SRID potency antibodies for pandemic influenza vaccines. Vaccine.

[B9] Khurana, S, Verma, S, Verma, N, Crevar, CJ, Carter, DM, Manischewitz, J, King, LR, Ross, TM, Golding, H (2010). Properly folded bacterially expressed H1N1 hemagglutinin globular head and ectodomain vaccines protect ferrets against H1N1 pandemic influenza virus. PLoS One.

[B10] Khurana S, Verma S, Verma N, Crevar CJ, Carter DM, Manischewitz J, King LR, Ross TM, Golding H (2011b). Bacterial HA1 vaccine against pandemic H5N1 influenza virus: evidence of oligomeriza-tion, hemagglutination, and crossprotective immunity in ferrets. J. Virol.

[B11] Lin YJ, Deng MC, Wu SH, Chen YL, Cheng HC, Chang CY, Lee MS, Chien MS, Huang CC (2008). Baculovirus derived hemagglutinin vaccine protects chickens from lethal homologous virus H5N1 challenge. J. Vet. Med. Sci.

[B12] Olsen, B, Munster, VJ, Wallensten, A, Waldenstrom, J, Osterhaus, ADME, Fouchier, RAM (2006). Global patterns of influenza A virus in wild birds. Science.

[B13] Shen S, Mahadevappa G, Oh HL, Wee BY, Choi YW, Hwang LA, Lim SG, Hong W, Lal SK, Tan YJ (2008). Comparing the antibody responses against recombinant hemagglutinin proteins of avian influenza A (H5N1) virus expressed in insect cells and bacteria. J. Med. Virol.

[B14] Shoji, Y, Bi, H, Musiychuk, K, Rhee, A, Horsey, A, Roy, G, Green, B, Shamloul, M, Farrance, CE, Taggart, B, Mytle, N, Ugulava, N, Rabindran, S, Mett, V, Chichester, JA, Yusibov, V (2009). Plant-derived hemagglutinin protects ferrets against challenge infection with the A/Indonesia/05/05 strain of avian influenza. Vaccine.

[B15] Shoji Y, Chichester JA, Jones M, Manceva SD, Damon E (2011). Plant based rapid production of recombinant subunit hemagglutinin vaccine targeting H1N1 and H5N1 influenza. Hum. Vaccines.

[B16] Stevens, J, Blixt, O, Tumpey, TM, Taubenberger, JK, Paulson, JC, Wilson, IA (2006). Structure and receptor specificity of the hemagglutinin from an H5N1 influenza virus. Science.

[B17] Swayne DE, Suarez DL (2000). Highly pathogenic avian influenza. Rev. Sci. Tech.

[B18] Tonegawa, K, Nobusawa, E, Nakajima, K, Kato, T, Kutsuna, T, Kuroda, K (2003). Analysis of epitope recognition of antibodies induced by DNA immunization against hemagglutinin protein of influenza A virus. Vaccine.

[B19] Treanor JJ, Campbell JD, Zangwill KM, Rowe T, Wolff M (2006). Safety and immunogenicity of an inactivated subvirion influenza A (H5N1) vaccine. N. Engl. J. Med.

[B20] Tsai HJ, Chi LA, Yu AL (2012). Monoclonal antibodies targeting the synthetic peptide corresponding to the polybasic cleavage site on H5N1 influenza hemagglutinin. J. Biomed. Sci.

[B21] Verma S, Dimitrova M, Munjal A, Fontana J, Crevar CJ, Carter DM, Ross TM, Khurana S, Goldinga H (2012). Oligomeric recombinant H5 HA1 vaccine produced in bacteria protects ferrets from homologous and heterologous wild-type H5N1 influenza challenge and controls viral loads better than subunit H5N1 vaccine by eliciting high-affinity antibodies. J. Virol.

[B22] Wei C, Nurul T, Wahida AG, Shaharum S (2014). Construction and heterologous expression of a truncated haemagglutinin (HA) protein from the avian influenza virus H5N1 in Escherichia coli. Trop. Biomed.

